# The Effect of Concentration Factor on Membrane Fouling

**DOI:** 10.3390/membranes7030050

**Published:** 2017-09-01

**Authors:** Appana Lok, Pierre R. Bérubé, Robert C. Andrews

**Affiliations:** 1 Department of Civil Engineering, University of Toronto, Toronto, ON M5S 1A4, Canada; andrews@ecf.utoronto.ca; 2 Department of Civil Engineering, Universityof British Columbia; Vancouver, BC V6T 1Z4, Canada; berube@civil.ubc.ca

**Keywords:** ultrafiltration, bench-scale, membrane fouling, concentration factor

## Abstract

Bench-scale systems are often used to evaluate pretreatment methods and operational conditions that can be applied in full-scale ultrafiltration (UF) systems. However, the membrane packing density is substantially different in bench and full-scale systems. Differences in concentration factor (CF) at the solution–membrane interface as a result of packing density may impact the mass transfer and fouling rate and the applicability of bench-scale systems. The present study compared membrane resistance when considering raw water (CF = 1) and reject water (also commonly referred to as concentrate water) (CF > 1) as feed in UF systems operated in deposition (dead-end) mode. A positive relationship was observed between the concentration of the organic matter in the solution being filtered and resistance. Bench-scale trials conducted with CF = 1 water were more representative of full-scale operation than trials conducted with elevated CFs when considering membrane resistance and permeate quality. As such, the results of this study indicate that the use of the same feed water as used at full-scale (CF = 1) is appropriate to evaluate fouling in UF systems operated in deposition mode.

## 1. Introduction

Hollow fiber ultrafiltration (UF) systems are increasingly being employed in drinking water treatment applications because of their ability to produce high-quality water and reduce the footprint of a facility [1]. The main drawback of UF—the focus of this study—is membrane fouling, which reduces productivity and increases operational costs of the systems [1]. Membrane fouling is largely attributed to organic fouling by biopolymers [2]. A number of studies have investigated pretreatment methods and operational conditions to reduce membrane fouling [1]. Much of this work has been based on the use of bench-scale systems that incorporate single membrane fibers [3,4,5,6], and as such have substantially different loading characteristics when compared to full-scale systems—in particular, the specific membrane surface area or packing density within a given system volume. As a result, most bench-scale studies may have been performed at concentration factors (CFs) that are much lower than those typical of full-scale systems. Considering that fouling is impacted by the concentration of the constituents in the solution being filtered, it is possible that the outcomes of bench-scale studies are not representative of those at full-scale—especially considering that the CF in most bench-scale studies is approximately 1 [2,6]. However, when operating in deposition mode (dead-end mode, no turbulence induced onto the membrane by cross-flow or air sparging during permeation), the CF in full-scale systems are theoretically not expected to change during a filtration cycle [7]. Unfortunately, limited information exists on the impact of CF on the outcomes of bench-scale studies. The motivation of the present study was to assess the impact of concentration factor on fouling (i.e., change in membrane resistance over time) at bench-scale, and compare to full-scale systems operated in deposition mode.

## 2. Materials and Methods

**Figure 1:**
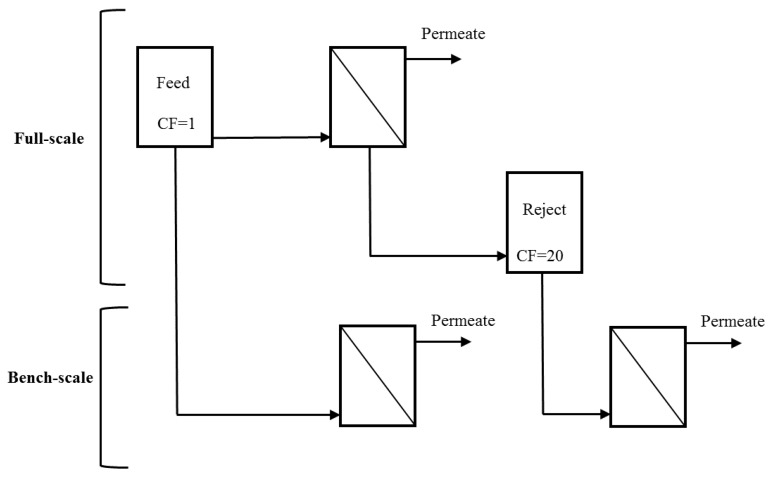
Schematic of the experiments in this study. Theoretical concentration factor (CF) values for membranes operated at 95% recovery are shown.

UF feed and reject water (obtained at the end of a permeation cycle following air sparging and backwashing) were collected from two full-scale water treatment plants (WTPs) during a single sampling event and used as influent to a bench-scale system [4,6] (Figure 1). The reject water corresponds to the maximum bulk CF that would be expected in the full-scale system. The reject water for membranes operated at 95% recovery is theoretically expected to have a CF of 20 (Equation (1)). Because the systems are operated in deposition mode, the theoretical concentration of foulants in the bulk liquid is expected to be equivalent to that in the feed [7]. However, the actual concentration of foulants at the membrane surface is expected to be greater than in the feed (Equation (2)).

(1) $$\mathrm{Theoretical}\;\mathrm{CF}=\frac{1}{1-\mathrm{percent}\;\mathrm{recovery}\left(\%\right)}$$

(2) $$\mathrm{Experimental}\;\mathrm{CF}=\frac{C_{m}}{C_{f}}$$

where: *C*_m_ = concentration of dissolved or particulate organics on the feed side of the membrane or reject water; *C*_f_ = concentration of dissolved or particulate organics in the feed water.

The membrane resistances measured when filtering UF feed water and reject water at bench-scale over a period of 24 h were compared (Equation (3)). Results obtained at bench-scale were also directly compared to full-scale systems (at the time when the feed and reject water were collected). Resistance due to fouling was quantified using Equation (3), which is the difference between the measured resistance (${\left(\frac{\Delta P}{J\mu }\right)}_{T}$) [8,9] and the intrinsic resistance (${\left(\frac{\Delta P}{J\mu }\right)}_{0}$) of the membrane at the start of each filtration test [10].

(3) $$R={\left(\frac{\Delta P}{J\mu }\right)}_{T}-{\left(\frac{\Delta P}{J\mu }\right)}_{0}$$

where: *R* is the normalized resistance (m^−1^), Δ*P* is the transmembrane pressure (Pa), *J* is the flux (m/s), and μ is the fluid viscosity(kg/m·s) at the test temperature.

The Lakeview WTP (Mississauga, ON, Canada) treats Lake Ontario (2 mg/L dissolved organic carbon (DOC), pH of 8.5) water using ozonation (typically 1 mg/L [11]), biofiltration, ultrafiltration, UV, and chlorination. The Barrie Surface WTP (Barrie, ON, Canada) treats Lake Simcoe water (4 mg/L DOC, pH of 8.4) using coagulation (4 mg/L polyaluminium chloride), ultrafiltration, granular activated carbon, and chlorination. Both plants operate their membranes in deposition mode with 95% recovery and use ZeeWeed^®^ 1000 (GE Water & Process Technologies, Oakville, ON, Canada), which are outside-in, polyvinylidene difluoride (PVDF) hollow fiber UF membranes. The membrane has a nominal pore size of 0.02 μm and pure water permeability of 1.48 L·m^−2^·h^−1^·kPa^−1^. A chemical recovery clean was performed on the full-scale system prior to data collection. Chemical cleans at the Barrie Surface WTP involve soaking the membranes in 500 mg/L of sodium hypochlorite followed by citric acid at a pH of approximately 3.5. At the Lakeview WTP, two types of chemical cleans are performed. Hypochlorite cleans (500 mg/L of sodium hypochlorite and sodium hydroxide at a pH of 11.3) and citric acid cleans (2000 mg/L citric acid and sulphuric acid at a pH of 2.1) are conducted every 30 and 60 days, respectively.

The single-fiber bench-scale system used in this study was similar to that previously described [6,12]. The system was specifically designed to mimic the hydrodynamic conditions and filtration/backwash cycles present at full-scale systems. The permeation and backwash cycles were 30 and 3 min, respectively. Conditions that mimic air sparging—used to induce turbulence onto the membranes—were only applied during backwash (permeation in deposition mode). A 25-cm length of virgin outside-in, hollow fiber UF membrane (ZeeWeed^®^ 500, GE Water and Process Technologies, Oakville, ON, Canada) (approximately 1250 mm^2^ of permeable area) was used for each filtration test. The fiber has a nominal pore size of 0.04 μm and pure water permeability of 2.15 L·m^−2^·h^−1^·kPa^−1^. Fibres were first soaked in 750 mg/L sodium hypochlorite solution for 24 h to remove residual shipping preservative and then stored in a 50 mg/L sodium hypochlorite solution until use. Immediately prior to the experiments, fibers were cleaned by filtering 750 mg/L sodium hypochlorite solution twice for 1 h each followed by Mille-Q water for 2 h, during which the clean water resistance of the virgin, loose membrane was quantified. The loose membrane was then mounted onto a stainless steel holder which was recessed into the wall of the membrane tank and secured using silicone. Milli-Q water was then filtered through the fiber for an additional 2 h. The change in resistance between the loose and mounted fibers was used to estimate the actual permeable membrane area. The permeate flow was monitored using a scale (Cole Parmer Symmetry Topbalance, Montreal, PQ, Canada) and adjusted to 50 L/m^2^∙h (i.e., identical to the flux at full-scale).

Feed and permeate samples were collected every 8 h during the 24-h filtration tests and analysed for total organic carbon (TOC) using an O-I Corporation Model 1010 TOC Analyser with a Model 1051 Vial Multi-Sampler (College Station, TX, USA), based on Standard Method 5310D [13]. Samples for dissolved organic carbon (DOC) were filtered using a 0.45 μm filter (Gelman Supor, Gelman Sciences, Ann Arbor, MI, USA) prior to analysis. Particulate organic carbon (POC) was calculated by subtracting DOC from TOC.

## 3. Results and Discussion

The DOC concentration in the UF reject water collected from the Barrie Surface WTP was twice that of the feed water (Table 1). However, the POC concentration in the UF reject water was 7.5 times greater. The DOC and POC concentrations in the UF reject water at the Lakeview WTP increased by a factor of 2.6 and 2.7, respectively. The POC concentration in the reject water for both systems was not 20 times greater than in the feed, as would be anticipated based on a 95% recovery rate.

**Figure 2:**
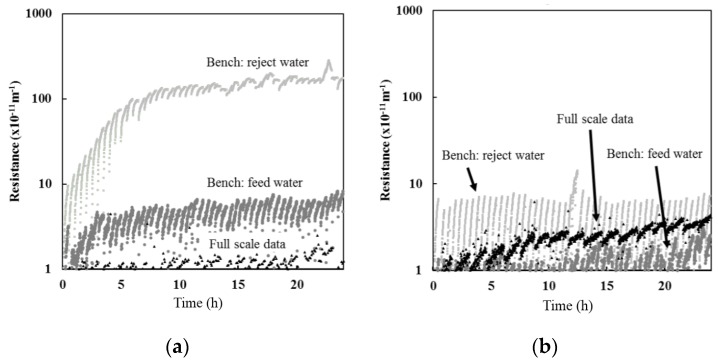
Results from filtration tests: (**a**) Barrie Surface WTP; and (**b**) Lakeview WTP. Full-scale resistance data are plotted for comparison.

**Figure 3:**
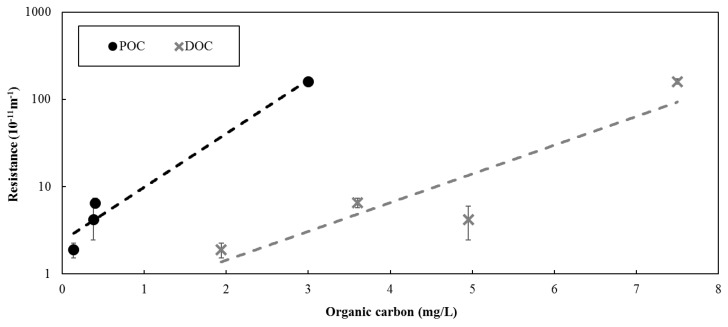
Resistance (averaged over the entire last permeation cycle) for each of the bench-scale filtration tests vs. organic carbon concentration (POC, DOC) in the solution filtered. The error bars represent the standard error associated with the resistance of the last permeation cycle. Exponential relationship was fitted to the data presented to illustrate the overall trend.

Bench-scale filtration results indicated that the resistance observed with reject water was greater than when using feed water for both Barrie Surface and Lakeview WTP (Figure 2). However, the difference between the results were more pronounced for the Barrie Surface WTP. The greater difference between the resistance observed between feed and reject water for the Barrie Surface WTP could be attributed to the higher concentration of organic material. As illustrated in Figure 3, a positive relationship was observed between the concentration of organic matter in the solution being filtered and the resistance. As such, the results suggest that the concentration of the solution being filtered largely determines the membrane resistance, which is consistent with previous studies [14,15].

While feed water organic carbon was not measured at full-scale for the data presented, the POC and DOC at full-scale ranges between 0.2–0.4 mg/L and 3.4–4 mg/L, respectively, at Barrie Surface WTP and 0.1–0.2 mg/L and 1.8–2.2 mg/L, respectively, at Lakeview WTP. Based on the relationships presented in Figure 3, the full-scale resistance is expected to range between 3.2–6.5 × 10^−11^ m^−1^ for Barrie Surface WTP, which is slightly above the actual full-scale resistance presented in Figure 2. The expected full-scale resistance for Lakeview WTP ranged from 1.2–3.2 × 10^−11^ m^−1^, which is consistent with actual full-scale resistance (Figure 2).

Results for both Barrie and Lakeview WTP suggest that bench-scale studies using full-scale feed water (CF = 1) is more representative of the actual resistance obtained at full-scale when compared to using full-scale reject water (CF > 1) (Figure 2).

When filtering feed water, the permeate quality in terms of DOC and POC was similar at bench and full-scale (Table 2). However, when filtering reject water at bench-scale, the permeate DOC was significantly greater than observed at full-scale. Similar to the resistance data, results for both plants suggest that bench-scale studies using full-scale feed water (CF = 1) is more representative in terms of the rejection of organic matter when compared to using full-scale reject water (CF > 1).

## 4. Conclusions

The impact of membrane packing density and the associated concentration factor on the fouling rate and the applicability of bench-scale systems was evaluated in this study. The expected elevated concentration at the membrane surface in densely packed or full-scale systems does not appear to largely influence resistance in UF systems operated in deposition mode. The results of the present study confirm that using full-scale feed water (CF = 1) in bench-scale studies to evaluate membrane fouling is valid.

## Figures and Tables

**Table 1:** Dissolved and particulate organic carbon (DOC and POC) concentration in feed and reject water collected from Barrie and Lakeview water treatment plants (WTPs).

Sampling Location	DOC (mg/L)			POC (mg/L)		
	Feed	Reject	CF	Feed	Reject	CF
Barrie WTP	3.6 ± 0.1	7.5 ± 0.1	2.1	0.40 ± 0.06	3.0 ± 0.07	7.5
Lakeview WTP	1.9 ± 0.1	5.0 ± 0.1	2.6	0.14 ± 0.08	0.38 ± 0.06	2.7

**Table 2:** Permeate DOC and POC concentrations at full and bench-scale.

Permeate Sample		Barrie WTP		Lakeview WTP	
		DOC	POC	DOC	POC
Full-scale		3.6 ± 0.2	0.11 ± 0.06	1.8 ± 0.2	0.05 ± 0.06
Bench-scale	Feed water	3.6 ± 0.2	0.10 ± 0.08	1.9 ± 0.2	0.07 ± 0.05
	Reject water	4.1 ± 0.3	0.10 ± 0.07	2.2 ± 0.2	0.05 ± 0.06
